# Hospital Festivals and Meetings

**Published:** 1895-05-25

**Authors:** 


### HOSPITAL FESTIVALS AND MEETINGS.

### NORTH-EASTERN HOSPITAL FOR CHILDREN, SHOREDITCH.

The annual meeting of the governors of this hospital was held in Devonshire House, Bishopsgate Street, on May 15th, Mr. J. Lister Godlee, the honorary treasurer, presided. The annual report was submitted. It showed that during the year there had been 701 in-patients. In the out-patient department there had been 13,124 new cases, a decrease on previous years, due to the closing of the out-patient department for several weeks while necessary repairs were carried out. Notwithstanding that the ordinary expenditure in 1894 had been £410 less than in 1893—a saving due in large measure to the fact that no festival dinner had been held—there was a deficit on the year's working of £362. The extraordinary expenditure had, however, been exceptionally heavy, the principal item being the alterations in the out-patient department. In January, 1894, it had been unfortunately necessary to obtain a further loan of £500, which increased the mortgage upon the hospital to £4,500. This was a heavy burden upon the resources of the hospital, and the committee made an earnest appeal for funds to enable them to clear it off. To provide a sufficient income an increase of £500 per annum in the annual subscriptions was essential. The Chairman, in moving the adoption of the report, emphasised the appeal that had been made for funds to clear off the mortgage, and dwelt upon the value of the work which the hospital had done and was doing. The report was adopted, and the usual votes of thanks to the committee, the medical staff, and the chairman were passed.

### INFANT ORPHAN ASYLUM, WANSTEAD.

Sir Algernon Borthwick, M.P., presided at the anniversary festival of this charity last week, held at the Haberdashers' Hall. Before the proposal of the toast, "The Infant Orphan Asylum," some hundred children of ages from three to fifteen marched round the hall in procession, and took up their position behind the guests, one of them, a little girl of eleven, delivering an address. Sir Algernon, in urging the wants of the asylum, said he thought that of all the causes of charity which could be pleaded none was more touching than that of children. There could be no nobler work than the endeavour to make the lives of such children happy, to give them education, and so fit them for the work of life. The chairman afterwards took occasion to extol the voluntary system of supporting charities, of the success of which this orphan asylum was so striking a product. Subscriptions to the amount of £1,508 were announced during the evening.

### ROYAL MEDICAL BENEVOLENT COLLEGE, EPSOM.

At the twenty-seventh festival dinner, in aid of the funds of the above institution (held on the 15th inst. at the Imperial Institute, the Right Hon. A. J. Balfour, M.P., in the chair), subscriptions and donations amounting to close on £1,700 were announced.

Sir Richard Webster, Q.C., M.P., in proposing the toast of "The Medical Corporations," said that the Royal Colleges of Physicians and of Surgeons were to him a most interesting study, in that they were the only two great corporations with which he was acquainted that had hitherto not been attacked or marked out for demolition or disestablishment. (Laughter.) And yet, as far as he could see, they possessed all the qualifications which should lead to such an attempt being made. They had each of them been established for upwards of four hundred years—(laughter)—they were, he believed, fairly wealthy—(laughter)—and they certainly had done most useful work. (Hear, hear.) Those were apparently the principal qualifications which in the present day led to an attempt at disestablishment. (Hear, hear, and laughter.) He had sought carefully for some reasons why these two institutions should enjoy this immunity from attack, and had come to the conclusion that the people who were likely to attack them were divided into two classes—those who were inspired by fear and those who were actuated by gratitude; those who were inspired by the fear that some day they would come under the hands of some medical man who, if they had not behaved themselves, would promptly proceed to take it out of them—(laughter)—and those who felt, as he (the speaker) felt, real gratitude for the noble services rendered by the members of the medical profession. (Applause.)

Mr. Christopher Heath (president of the Royal College of Surgeons) and Sir Dyce Duckworth (in the absence of the president of the Royal College of Physicians) responded on behalf of their corporations. The latter said that the physicians of England had a real sympathy with the magnificent institution whose welfare they had assembled there that evening to promote. (Applause.)

Dr. E. Long Fox, president of the British Medical Association, who also responded, speaking for the general practitioners (than whom, he said, no body of Her Majesty's subjects won more affection or devoted themselves with more self-denial to the well-being of their fellow-men), said that the Medical Benevolent College was valued by them not only for the education it gave; not only for the fact that that education was given to the sons of men, often of great ability, often very hard-working, whose life had been placed under circumstances where devotion and skill could not be recompensed adequately; but because they felt that the children educated within the walls of that medical college were imbued with two ideas—one, self-sacrifice for the sake of others, and the other, the highest possible idea of professional tone. (Applause.)

The Chairman, in proposing "Success to the Royal Medical Benevolent College," said it was probably not necessary for him to describe to those present the characteristics of the great Medical Benevolent College which stood at the present time so urgently in need of assistance. Let it suffice, then, for him to briefly remind them that it was intended to carry out the double function of giving pensions to those members of the profession (or their widows) who most deserved it, and of supplying the best possible education for their children. That such an institution was useful, nay, that it was necessary, he imagined few would be disposed to deny—(hear, hear)—and that the medical men of the United Kingdom, numbering as they did some 32,000 persons, should have such an institution to serve the needs of their less fortunate members, was what would have been expected. (Hear, hear.) Appealing as he was to the general public on behalf of that college, the question naturally and necessarily arose, why this great body of 32,000 persons should look outside their own limits in order to obtain assistance for a charitable institution whose beneficent efforts were necessarily confined to their own body? The answer to that question was an easy one, and it was to be found in the obligations which the outside public, in whose name he ventured to speak, and one of whom he was, were under, and the benefits they had derived from the medical profession. (Hear, hear.) There was no body of men, select them how one might or where one might, who had given to the public to which he now appealed a larger measure of gratuitous service. (Hear, hear.) In every district, in every parish, almost in every street, it would be found that the medical profession had ever been ready to go, with or without remuneration, to the succour of the unfortunate—(hear, hear)—and that they had lavished the treasures of their time and of their skill on those who were from their worldly circumstances but very ill able to repay them. (Hear, hear.) When, therefore, it was remembered that the great charity on whose behalf he was speaking was not called into existence merely to subserve the interests of the incompetent or the unskilful members of the medical profession, but, on the contrary, the interests of those frequently occurring cases where a medical practitioner, from no want of skill, certainly from no lack of energy, zeal, or public spirit, had carried out his great work in districts and under circumstances which made him not indeed less, but, it might be, more useful to mankind, and which made it quite impossible for him to leave an adequate provision for his wife and children. (Applause.) These latter members of the profession (not the waifs and strays, not the wastage which must necessarily occur in so vast a body as that to which he referred), men who were very often prominent members of the body in point of zeal and devotion, were the people whose interests that charity subserved. (Applause.) He felt sure the public would, therefore, feel that they had a duty to perform in connection with this college, and that among the great charities of the country calling for their support the Medical Benevolent College did not rank among the least. (Applause.)

Dr. C. Holman, treasurer of the institution, said that on every New Year's Day he stood in the position of having during the next twelve months to find £6,000, while he could depend only upon an income of something like £800. In the course of his speech he paid tribute to the aid which had been extended to the college by the Press, and especially by Mr. Burdett, Dr. Dawson Williams, and Mr. T. H. Wakley.

The Rev. T. N. Hart Smith, the head master, who also replied, said that they endeavoured at Epsom College to turn out boys who were not fast on the one hand, nor prigs on the other. At the present moment there were on the staffs of the London hospitals no less than seven former pupils, and during the last few years 14 open scholarships at the University had been won by boys from Epsom College.

### THE HOSPITAL FOR SICK CHILDREN.

In securing for the second year in succession a member of the Royal Family to preside at their anniversary festival the Committee of the Hospital for Sick Children in Great Ormond Street were peculiarly favoured. This year's function took place on Friday in last week at the Hotel Metropole, and among the company assembled under the presidency of the Duke of Cambridge were: His Grace the Duke of Fife, the Bishop of Bath and Wells, the Right Hon. Lord Kinnaird, Lord Monkswell, Sir R. Quain, Bart., M.D., Dr. Barlow, and Mr. Adrian Hope (secretary).

The preliminaries having been somewhat quickly disposed of, the toast of "Prosperity to the Hospital for Sick Children" was submitted by the Chairman. He had visited the hospital, and was able to vouch that he had never been in an institution so clean, so well organised, and so well arranged. Everything he saw reflected the greatest credit on the management, the nurses, and the medical officers, and he was sure the money spent on that valuable and useful institution was wisely spent. Referring to the progress made by the hospital, the Duke mentioned that the in-patients had increased from 1,281 in 1892, to 1,678 in 1894, and the out-patients from 21,045 to 27,334. Coupled with the toast was the name of the President of the hospital.

The Duke of Fife, in responding, said there was no good and useful cause in this country which the chairman was not ready to assist, and he thanked him for the very prompt and willing manner in which he consented to take the chair that evening. There were many good causes in this country that enlisted sympathy and help, but there were none more deserving than the Hospital for Sick Children, for while other sufferers could help themselves a sick child was peculiarly helpless and dependent upon others. One of the best friends of the hospital was Charles Dickens. He took the chair at the first dinner (in 1858), and certainly no one in the whole branch of literature had ever described in more eloquent or touching words the needs and the wants and the suffering of little children; and the most pathetic and powerful appeal ever made in aid of that hospital came from the great novelist, whose name ought to be cherished by every child in this country. (Applause.)

Lord Monkswell, as a member of four classes who were presumed to be able to speak on any subject at any time to any person, viz., politicians, peers, barristers, and county councillors, supposed it was on that assumption he was asked to take charge of the toast of "The Committee of Management." Coupled with it was the name of Mr. Arthur Lucas, the chairman of the committee, and Lord Monkswell testified to the able manner in which Mr. Lucas gave evidence before the House of Lords' Committee on Metropolitan Hospitals. Every member of the committee felt that he was so imbued with all the details of hospital management that he must have given an immense amount of time and labour to the duties he was called upon to discharge.

Mr. Lucas, in his reply, took occasion to thank the chairman on behalf of the committee for coming there that night, and said it was generally known that the Duke, when he was invited had closed the list of charities he intended to attend this season, but reopened it and included that hospital. After giving some statistics of the work of the hospital last year Mr. Lucas mentioned that at one time a fourth of the staff was incapacitated by influenza. The remainder of the staff, however, stuck to their work well, and they never had to refuse a single patient. (Hear, hear.)

The toast of the "Medical Officers" was submitted by the Bishop of Bath and Wells, who drew particular attention to the fact that only 245 deaths took place last year among the 1,671 in-patients. Dr. Edmund Owen replied. Some time since, he said, there was a boy in the hospital from whom the house-surgeon had to remove every morning a painful dressing, and every morning the boy cried. "Why cry?" the nurse asked, and the boy replied, "I know it must be done, but if I cry it makes the doctor more careful." (Laughter.) As a matter of fact they dealt with all those children as if the patient's eyes were brimming over with pathetic tears. They dealt with the sick children exactly as in similar circumstances they would like their own dear ones to be dealt with. (Applause.)

The proceedings closed with the announcement from the secretary of contributions to the extent of £2,132.

